# Antibacterial and antitumour activities of some plants grown in Turkey

**DOI:** 10.1080/13102818.2014.909708

**Published:** 2014-07-08

**Authors:** Canan Usta, Arzu Birinci Yildirim, Arzu Ucar Turker

**Affiliations:** ^a^ Department of Biology, Gaziosmanpasa University, Tokat, Turkey; ^b^ Department of Biology, Abant Izzet Baysal University, Bolu, Turkey

**Keywords:** antibacterial, antitumour, pathogenic bacteria

## Abstract

Screening of antibacterial and antitumour activities of 33 different extracts prepared with three types of solvents (water, ethanol and methanol) was conducted. The extracts were obtained from 11 different plant species grown in Turkey: *Eryngium campestre* L., *Alchemilla mollis* (Buser) Rothm., *Dorycnium pentaphyllum* Scop., *Coronilla varia* L., *Onobrychis oxyodonta* Boiss., *Fritillaria pontica* Wahlenb., *Asarum europaeum* L., *Rhinanthus angustifolius* C. C. Gmelin, *Doronicum orientale* Hoffm., *Campanula glomerata* L. and *Campanula olympica* Boiss. Antibacterial activity against six bacteria was evaluated: *Escherichia coli*, *Pseudomonas aeruginosa*, *Klebsiella pneumoniae*, *Streptococcus pyogenes*, *Staphylococcus aureus* and *Staphylococcus epidermidis* by using disc diffusion and well diffusion methods. *S. aureus* and *S. epidermidis* were most sensitive to the methanolic extract from *A. europaeum*. *S. pyogenes* was vulnerable to all used extracts of *D. orientale*. In addition, ethanolic or methanolic extracts of *E. campestre*, *A. mollis*, *D. pentaphyllum*, *C. varia*, *R. angustifolius*, *C. glomerata* and *C. olympica* displayed strong antibacterial activity against at least one of the tested gram-negative bacteria. The methanolic extract from *R. angustifolius* showed a broad-spectrum activity against both gram-positive and gram-negative bacteria. Antitumour activity was evaluated with *Agrobacterium-tumefaciens*-induced potato disc tumour assay. Best antitumour activity was obtained with the aqueous extract from *A. europaeum* and methanolic extract from *E. campestre* (100% and 86% tumour inhibition, respectively).

## Introduction

Plants contain thousands of constituents and are a valuable source of new and biologically active molecules. In order to discover new bioactive compounds from plant sources that could become new leads or new drugs, extracts should be simultaneously evaluated by chemical screening and by various biological or pharmacological targets.[1] Biological screening is necessary to provide a scientific basis for validation of the traditional utilization of medicinal plants. Preclinical biological screening is important not only for establishing the therapeutic efficacy of the medicinal plants but also to validate their historical utilization by traditional healers and herbalists. This is especially important since the plants may have evolved over a period of time leading to changes in their chemical composition and thus the biological activity. Preclinical studies allow comparison of efficacy of different plants and help in designing of rational drug combinations.[2]

*Eryngium* spp. have been used in folk medicine as antispasmodic, aromatic, diaphoretic, diuretic, expectorant, stimulant, nervine and aphrodisiac.[3] *Eryngium campestre* has anti-inflammatory and antinociceptive activities.[4] *E. campestre* includes saponins,[5] coumarin,[6] monoterpene glycosides [7] and flavonoids.[8] *Alchemilla* spp. are rich in tannin and so are an effective astringent and styptic, commonly used both internally and externally in the treatment of wounds.[9] They have a long history of herbal use, mainly as an external treatment for cuts and wounds, and internally in the treatment of diarrhoea and a number of women's ailments, especially menstrual problems.[10] High level of anti-inflammatory activity of *Dorycnium pentaphyllum* was recorded.[11] *Coronilla varia* is a cardiotonic and seeds of this plant have antitumour activity due to their cardenolide content.[12,13] High antioxidant activity of acetone and methanol extracts of aerial parts of sainfoin (*Onobrychis viciifolia*) was recorded.[14] Ethanolic extracts of *Fritillaria pontica* fruits have high antioxidant activity.[15] *Asarum eurapeum* contains phenylpropanoids leading to local anesthetic and expectorant activities.[16] Toth et al. [17] described the flavonoids of *Rhinanthus angustifolius*. Traditionally, *Rhinanthus minor* has been used to treat eye complaints (ophthalmic).[18] *Doronicum* spp. are useful in the treatment of nervous depression. *Campanula* spp. have been traditionally used for all inflammation of the mouth and throat.[9]

The aim of this study was to evaluate the antibacterial and antitumour activities of 11 plant species found in Bolu, Turkey.

## Materials and methods

### Plant material and extraction

Aerial parts of plants including flowers, leaves and stems were collected from Abant Lake, Bolu, Turkey. Identification of species was made by using ‘Flora of Turkey and the East Aegean Islands’ [19] and voucher specimens were deposited at the Abant Izzet Baysal University (AIBU) Herbarium, Bolu, Turkey.

All collected plants were oven dried at 40 °C for a week and extracted with different solvents: water, methanol (MeOH) and ethanol (EtOH). For aqueous extraction, 20 g from each powdered plant sample were extracted with 200 ml water at 80 °C in a waterbath for 12 hs and then filtered. Water was evaporated using a lyophilizator. For alcoholic extractions (MeOH and EtOH), 20 g from each powdered plant sample were soxhlet extracted with 350 ml MeOH or EtOH at 60 °C for 12 h and liquid portion was evaporated by rotary evaporator. For antibacterial and antitumour assays, residue was dissolved in sterile distilled water in order to obtain a final concentration of 100 mg/ml. All extracts were sterilized by filtering through a 0.22 μm filter (Millex). Plant materials, designation of treatments and yield (%) for each extraction are summarized in Table 1.

**Table 1.** Designation of studied plant extracts, their family and botanical names, used parts and collection numbers.

Family and plant species	Collection number	Extract	Designation	Yield (%)*
APIACEAE		Water	1X	1.72
*Eryngium campestre* L.	AUT-2018	EtOH	1Y	1.80
var. *virens* Link.		MeOH	1Z	2.20
ROSACEAE		Water	2X	2.00
*Alchemilla mollis* (Buser) Rothm.	AUT-2019	EtOH	2Y	1.19
		MeOH	2Z	2.90
FABACEAE		Water	3X	2.20
*Dorycnium pentaphyllum* Scop.	AUT-2020	EtOH	3Y	5.60
subsp. *anatolicum* (Boiss.) Gams		MeOH	3Z	6.70
		Water	4X	3.30
*Coronilla varia* L.	AUT-2022	EtOH	4Y	7.01
subsp. *varia*		MeOH	4Z	8.13
		Water	5X	1.60
*Onobrychis oxyodonta* Boiss.	AUT-2026	EtOH	5Y	2.40
		MeOH	5Z	3.40
LILIACEAE		Water	6X	2.79
*Fritillaria pontica* Wahlenb.	AUT-2023	EtOH	6Y	1.94
		MeOH	6Z	6.35
ARISTOLOCHIACEAE		Water	7X	2.74
*Asarum europaeum L.*	AUT-2024	EtOH	7Y	1.57
		MeOH	7Z	4.00
SCROPHULARIACEAE		Water	8X	2.52
*Rhinanthus angustifolius* C.C. Gmelin	AUT-2025	EtOH	8Y	2.50
		MeOH	8Z	3.10
ASTERACEAE		Water	9X	4.10
*Doronicum orientate* Hoffm.	AUT-2021	EtOH	9Y	5.30
		MeOH	9Z	9.20
CAMPANULACEAE		Water	10X	2.95
*Campanula glomerata L.*	AUT-2027	EtOH	10Y	2.99
subsp. *Hispida* (Witasek) Hayek		MeOH	10Z	5.35
		Water	11X	1.97
*Campanula olympica* Boiss.	AUT-2028	EtOH	11Y	2.87
		MeOH	11Z	4.00

*Yield (%) = weight of extract (g)/20 g of powdered plant sample × 100

### Antibacterial assays

The bacteria used were as follows: *Streptococcus pyogenes* (ATCC 19615), *Staphylococcus aureus* (ATCC 25923) and *Staphylococcus epidermidis* (ATCC 12228) which are gram-positive bacteria, and *Escherichia coli* (ATCC 25922), *Pseudomonas aeruginosa* (ATCC 27853) and *Klebsiella pneumoniae* (ATCC 13883) which are gram-negative bacteria. The stock cultures were maintained by regular subculture to BHI broth (Merck) and then incubated at 37 °C overnight. This culture served as the inoculums for the susceptibility studies, starting with approximately 10^6^ CFU/ml in the test tubes. These colony-forming unit (CFU) counts were accurately and reproducibly obtained by inoculation of 0.1 ml of the culture having an absorbance value of 0.2 as determined by optical density measurement at 600 nm using an ultraviolet–visible spectrophotometer (Perkin Elmer Lambda 850, USA).

### Antibacterial screening

Thirty-three plant extracts were tested for their antibacterial activity. Two agar diffusion methods, well diffusion assay and disc diffusion assay were used to compare the susceptibility of the bacterial strains to the plant extracts.[20,21]

Well diffusion assay was used to provide semi-quantitative measures of antibacterial activity. Ten ml of top agar prepared with Muller Hinton Broth was seeded with 10^5^ CFU/ml of target bacteria and 0.1 ml of sterilized plant extracts were added to 6 mm diameter wells in the top agar previously prepared by using sterile pipette tips cut as 6 mm using micropipettes. One hundred μl of broth was added into wells to serve as negative controls. All plates were then incubated at 37 ºC for a period of 24 h. Then, the clearance zones around the wells (growth inhibition zones) were measured in millimetres. All experiments were repeated three times.

Kirby-Bauer disk diffusion test was performed on Mueller Hinton agar plates inoculated by using cotton swabs. Sterile filter paper discs (6 mm in diameter) were impregnated with 15 μl of extract. There were five replicates in each plate and two plates for each extract tested for each bacterium. Positive controls consisted of five different antimicrobial susceptibility test discs (Bioanalyse): Lincomycin (15 μg), Ampicillin (10 μg), Carbenicillin (100 μg), Tetracycline (30 μg), Bacitracin (10 U) and Novobiocin (30 μg). Broth was used as a negative control in the same controlling plates. Inoculated plates with discs were placed in a 37 °C incubator. After 16–18 h of incubation, inhibition zone diameter (mm) was measured. All experiments were repeated three times.

### Microscopic image assessment

The bacteria that were most susceptible to the plant extracts obtained by using different solvents were analysed topographically. The method was based on the scanning electron microscope (SEM) observations. For SEM analysis, small agar pieces were cut out from the inhibition zone and were fixed in 3.5% (v/v) glutaraldehyde in 0.1 M sodium phosphate buffer (pH 7.2) for half an hour at room temperature. They were then washed three times in the same buffer. The pieces were then postfixed in 1% (w/v) osmium tetroxide (OsO_4_) for an hour and then washed three times in the buffer. They were dehydrated in a graded alcohol series. Eventually, after the last dehydration with propylene oxide (CH_3_CH.CH_2_.O), the fixed material was then mounted on stubs using double-sided carbon tape and coated with gold/palladiumin sputter coater system in a high-vacuum chamber (Polaron SC7620, UK) for 150 s at 9 mA. The samples were examined and digital images captured using a JEOL JSM 5500 SEM at an accelerating voltage of 5 kV.

### Antitumour assay

Antitumour activity of all extracts was assessed with the potato disc method as modified by McLaughlin's group [22–24]. *Agrobacterium tumefaciens* (ATCC 23341) was cultured on yeast extract media (YEM) for 2–3 days at 28 °C. Camptothecin (Sigma) (tumour suppressant) served as a positive control and water was used as a negative control. Suspensions of *A. tumefaciens* in phosphate-buffered saline (PBS) were standardized to 1.0 × 10^9^ CFU as determined by an absorbance value of 0.96 ± 0.02 at 600 nm [22–24]. All extracts and control solutions were filter sterilized (sterile 0.22 μm filter, Millex). The test solutions consisted of 600 μl extract or control solution, 150 μl sterile distilled water and 750 μl of the standardized *A. tumefaciens* in PBS.

Potatoes (*Solanum tuberosum* L.) were washed and scrubbed with a brush under running water and surface sterilized by immersion in 10% commercial bleach (Domestos) for 20 min. Tubers were then placed on sterile paper towels and cut along either side revealing the largest surface area available. The trimmed tubers were then immersed in 20% commercial bleach (Domestos) for 15 min. Cylinders (10 mm diameter) were cut from the centre of potato tissue (skin portion was eliminated) using a cork borer on sterile paper towels and placed in sterile distilled water with lactic acid (pH = 4.0). Cylinders were rinsed twice more using sterile distilled water with lactic acid. Each cylinder was cut into 0.5 cm discs after excluding 1 cm end pieces. These discs were transferred to 24-well culture plates containing water agar (15 g/L). Each disc was overlaid with 50 μl of appropriate inoculum. No more than 30 min elapsed between cutting the potato discs and inoculation. Plates were incubated at 28 °C in the dark for two weeks. After two weeks, discs were stained with Lugol's reagent (I_2_KI; 5% I_2_ plus 10% KI in distilled water) and tumours on each disc were counted. Lugol's reagent stains the starch in potato tissue dark blue to dark brown colour, but the tumours do not take up the stain and appear creamy to orange. Experiments were repeated three times. Percent inhibition of tumours was calculated by using the following formula [22–24]:

% inhibition = [(solvent control mean – tested extract mean)/solvent control mean] × 100.

### Bacterial viability testing

Standardized bacterial suspension (1 × 10^9^ CFU of *A. tumefaciens* in PBS) was serially diluted with PBS to 1 × 10^3^ CFU. Bacterial viability was determined by incubating 1 ml of each plant extract with 1 ml of bacterial suspension (1 × 10^3^ CFU of *A. tumefaciens* in PBS) in microcentrifuge tubes (four tubes per extract) and left for 30 min. At 30 min after inoculation, 0.1 ml of inoculum (bacteria + extract) was removed and inoculated on YEM with spread plate technique. After 24 h incubation of inoculated plates at 28 °C, colony counts were made. Also, bacterial growth was evidenced by growth across the plates.[25]

### Data analysis

All data were analysed by analysis of variance (ANOVA) and mean values were compared with Duncan's Multiple Range Tests using SPSS ver. 15 (SPSS Inc., Chicago, IL, USA).

## Results and discussion

### Antibacterial activity

The solvents with their increasing order of polarity were used for the extraction of 11 different plants; these were ethanol, methanol and water. The percent yields of the extracts were shown in Table 1. Antibacterial activity of 33 different extracts prepared with three kind of solvents (water, methanol and ethanol) of 11 different plant species were studied by both the disc and well diffusion methods (Tables 2 and 3). Tested plant extracts showed similar antibacterial spectrum with both methods (Tables 2 and 3). Bacterial growth was generally sensitive to the reference antibiotics tested (Table 2). Inhibition zones varied from 36 mm for ampicillin and *S. epidermidis* to 7 mm for lincomycin and *P. aeruginosa* (Table 2). Since final concentrations of all extracts were adjusted with distilled water, it was used as a negative control and there was no inhibition with this control solvent.

**Table 2.** Antibacterial activity of used plant extracts (disc diffusion assay, means with the same letter within columns are not significantly different at *P* > 0.05).

	Mean diameter of inhibitory zones (mm ± SE)					
Treatments	*S. auerus*	*S. epidermidis*	*S. pyogenes*	*P. aeruginosa*	*K. pneumonias*	*E. coli*
1x	–	–	–	11.33 ± 0.66 fg	–	–
1y	–	11.33 ± 0.66 ef	13.33 ± 0.66 ij	20.00 ± 1.15 c	–	28.67 ± 1.33 a
1z	–	–	9.33 ± 0.66 mn	10.67 ± 0.66 g	–	17.33 ± 0.66 d
2x	–	**–**	–	–	–	9.33 ± 0.66 h
2y	–	–	–	22.67 ± 0.66 a	–	15.33 ± 0.66 de
2z	–	10.00 ± – fg	–	22.67 ± 1.33 a	**–**	9.33 ± 0.66 h
3x	14.67 ± 0.66 gh	**–**	18.67 ± 0.66 e	–	**–**	**–**
3y	18.00 ± 1.15 e	**–**	9.33 ± 0.66 mn	22.00 ± 1.15 ab	**–**	15.33 ± 1.33 de
3z	–	**–**	14.93 ± 0.33 hi	20.00 ± 1.15 c	**–**	13.00 ± 1.52 fg
4x	–	**–**	–	11.33 ± 0.66 fg	9.33 ± 0.66 j	13.33 ± 1.66 ef
4y	14.67 ± 1.33 gh	**–**	16.67 ± 1.33 fg	11.33 ± 0.66 fg	18.67 ± 0.66 bo	11.33 ± 0.66 g
4z	–	13.33 ± 0.66 d	–	19.33 ± 0.66 c	20.67 ± 0.66 a	–
5x	–	–	–	–	12.00 ± 1.15 i	–
5y	11.33 ± 0.66 j	13.33 ± 0.66 d	–	–	12.00 ± 0.00 i	**–**
5z	–	–	11.33 ± 0.66 kl	–	15.33 ± 0.66 ef	**–**
6x	–	–	15.33 ± 0.66 gh	8.67 ± 0.66 h	14.67 ± 1.33 fg	**–**
6y	–	–	–	13.33 ± 0.66 e	14.67 ± 0.66 fg	**–**
6z	16.67 ± 0.66 ef	–	–	17.33 ± 0.66 d	13.33 ± 0.66 ghi	15.33 ± 0.66 de
7x	22.00 ± 1.15 c	–	–	–	–	9.33 ± 0.66 h
7y	20.00 ± 1.15 d	–	–	–	12.67 ± 0.66 hi	17.33 ± 0.66 d
7z	24.00 ± 1.15 b	23.33 ± 0.66 c	–	11.33 ± 0.66 fg	12.67 ± 0.66 hi	16.00 ± 1.15 d
8x	12.67 ± 0.66 ij	9.33 ± 0.66 g	–	–	17.33 ± 0.66 cd	–
8y	13.33 ± 1.76 hi	–	12.67 ± 0.66 jk	13.33 ± 0.66 e	20.00 ± 1.15 ab	19.33 ± 0.66 c
8z	18.00 ± 1.15 e	8.67 ± 0.66 g	10.67 ± 0.66 Im	20.67 ± 0.66 be	21.33 ± 0.66 a	23.33 ± 0.66 b
9x	16.67 ± 0.66 ef	11.00 ± 2.08 ef	20.67 ± 0.66 cd	–	–	–
9	14.33 ± 0.33 ghi	12.33 ± 2.18 de	19.33 ± 0.66 de	–	**–**	13.33 ± 1.66 ef
9z	12.67 ± 0.66 ij	–	13.33 ± 1.66 ij	–	–	9.33 ± 0.66 h
10x	–	–	8.67 ± 0.66 n	–	12.00 ± 0.00 i	–
10y	–	–	10.67 ± 0.66 Im	–	17.33 ± 0.66 cd	**–**
10z	–	–	–	–	14.00 ± 0.00 fgh	**–**
11x	–	–	–	–	17.33 ± 0.66 cd	**–**
11y	–	–	12.00 ± 0.00 jkl	–	20.67 ± 0.66 a	13.33 ± 0.66 ef
11z	–	–	–	–	16.67 ± 0.66 de	**–**
Lincomycin	21.00 ± 0.00 cd	24.00 ± 0.00 c	12.00 ± 0.00 jkl	7.00 ± 0.00 i	12.00 ± 0.00 i	8.00 ± 0.00 h
Carbenicillin	18.00 ± 0.00 e	31.00 ± 0.00 b	25.00 ± 0.00 b	22.00 ± 0.00 ab	21.00 ± 0.00 a	21.00 ± 0.00 c
Ampicillin	26.00 ± 0.00 a	36.00 ± 0.00 a	31.00 ± 0.00 a	8.00 ± 0.00 h	17.00 ± 0.00 d	16.00 ± 0.00 d
Novobiocin	25.00 ± 0.00 ab	25.00 ± 0.00 c	17.00 ± 0.00 f	23.00 ± 0.00 a	12.00 ± 0.00 i	17.00 ± 0.00 d
Bacitracin	22.00 ± 0.00 c	14.00 ± 0.00 d	21.00 ± 0.00 c	11.00 ± 0.00 g	8.00 ± 0.00 j	9.00 ± 0.00 h
Tetracycline	16.00 ± 0.00 fg	24.00 ± 0.00 c	26.00 ± 0.00 b	13.00 ± 0.00 ef	14.00 ± 0.00 fgh	20.00 ± 0.00 c

**Table 3.** Antibacterial activity of used plant extracts (well diffusion assay, means with the same letter within columns are not significantly different at *P* > 0.05).

	Inhibition (mm ± SE)					
Treatments	*S. auerus*	*S. epidermidis*	*S. pyogenes*	*P. aeruginosa*	*K. pneumonia*	*E. coli*
1x	–	–	–	0.37 ± 0.03 fgh	–	–
1y	–	0.43 ± 0.03 b	0.47 ± 0.03 ef	0.73 ± 0.07 c	–	0.27 ± 0.03 kl
1z	–	–	0.27 ± 0.03 ij	0.40 ± 0.66 efg	–	0.70 ± 0.00 cd
2x	–	–	–	–	–	0.23 ± 0.03 I
2y	–	–	–	1.00 ± 0.06 a	–	0.63 ± 0.03 de
2z	–	0.43 ± 0.03 b	–	0.83 ± 0.03 b	–	0.27 ± 0.03 kl
3x	0.63 ± 0.03 def	–	0.83 ± 0.03 ab	–	–	–
3y	0.60 ± 0.06 def	–	0.20 ± 0.00 j	1.00 ± 0.06 a	–	0.57 ± 0.07 ef
3z	–	–	0.43 ± 0.03 fg	0.93 ± 0.03 a	–	0.43 ± 0.03 hi
4x	–	–	–	0.27 ± 0.03 i	1.83 ± 0.17 a	0.47 ± 0.03 gh
4y	0.53 ± 0.03 ef	–	0.63 ± 0.03 c	0.33 ± 0.07 ghi	0.77 ± 0.03 de	0.37 ± 0.03 ij
4z	–	0.47 ± 0.03 b	–	0.53 ± 0.03 d	1.00 ± 0.06 a	–
5x	–	–	–	–	0.37 ± 0.03 j	–
5y	0.33 ± 0.03 g	0.47 ± 0.03 b	–	–	0.47 ± 0.03 hij	–
5z	–	–	0.37 ± 0.07 gh	–	0.63 ± 0.03 efg	–
6x	–	–	0.57 ± 0.03 exi	0.30 ± 0.00 i	0.47 ± 0.03 hij	–
6y	–	–	–	0.47 ± 0.03 de	0.60 ± 0.00 fgh	–
6z	0.67 ± 0.03 de	–	–	0.80 ± 0.06 be	0.43 ± 0.03 ij	0.53 ± 0.03 fg
7x	0.93 ± 0.03 c	–	–	–	–	0.30 ± 0.00 jkl
7y	8.33 ± 0.33 a	–	–	–	0.47 ± 0.03 hij	0.57 ± 0.03 ef
7z	1.17 ± 0.03 b	1.07 ± 0.03 a	–	0.37 ± 0.03 fgh	0.67 ± 0.03 efg	0.73 ± 0.03 c
8x	0.47 ± 0.03 efg	0.27 ± 0.03 cd	–	–	0.77 ± 0.07 de	–
8y	0.47 ± 0.03 efg	–	0.53 ± 0.03 de	0.43 ± 0.03 ef	0.77 ± 0.03 de	0.87 ± 0.03 b
8z	0.67 ± 0.03 de	0.20 ± 0.66 e	0.37 ± 0.03 gh	0.77 ± 0.03 be	0.93 ± 0.07 be	1.27 ± 0.70 a
9x	0.77 ± 0.03 exi	0.30 ± 0.06 c	0.77 ± 0.03 b	–	–	–
9y	0.57 ± 0.03 ef	0.23 ± 0.03 de	0.87 ± 0.03 a	–	–	0.33 ± 0.03 jk
9z	0.43 ± 0.03 fg	–	0.33 ± 0.03 hi	–	–	0.27 ± 0.03 kl
10x	–	–	0.33 ± 0.03 hi	–	0.53 ± 0.03 ghi	–
10y	–	–	0.27 ± 0.03 ij	–	0.63 ± 0.03 efg	–
10z	–	–	–	–	0.57 ± 0.03 ghi	–
11x	–	–	–	–	0.63 ± 0.03 efg	–
11y	–	–	0.47 ± 0.03 ef	–	0.87 ± 0.03 exi	0.57 ± 0.07 ef
11z	–	–	–	–	0.73 ± 0.07 def	–

All used extracts showed inhibitory activity against at least one bacterial pathogen for both the disc and well diffusion methods (Tables 2 and 3). Especially, methanolic extract of *R. angustifolius* exhibited a broad-spectrum activity against both gram-positive and gram-negative bacteria (Tables 2 and 3). This activity against both types of bacteria may be indicative of the presence of broad-spectrum antibiotic compounds or simply general metabolic toxins. According to one record,[18] *R. minor* has been used traditionally to treat eye complaints. A broad-spectrum of antibacterial activity of *R. angustifolius* may explain why *Rhinanthus* spp. are used in folk medicine to treat eye conditions (caused by *S. aureus*, *S. epidermidis*, *S. pyogenes and P. aeruginosa*).

The gram-positive bacteria commonly seem to be more susceptible to the inhibitory effects of the plant extracts than the gram-negative bacteria do. Susceptibility of gram-positive bacteria may come from the possible inhibitory action of the components in this plant extract; the peptidoglycan layer is thicker than the gram-negative cell walls. On the contrary, *P. aeruginosa*, *K. pneumoniae* and *E. coli* which are gram-negative bacteria seemed to be more susceptible to used plant extracts in our experiments (Tables 2 and 3). Most importantly, subunits (lipopolysaccharides and lipoproteins) on the external cell membrane of gram-negative bacteria might well prevent the attachment of the components in some particular plant extracts from entering the cell. The variation of susceptibility of the tested microorganisms could be attributed to their intrinsic properties that are related to the permeability of their cell surface to the extracts. Their mechanisms of action may well be due to the disintegrity of the bacterial membranes. The extracts most probably have an effect on the action of proton motive force resulting into non-controlled electron flow. As a result, the aggregation of intracellular materials of the bacterial cell may occur.

With regard to gram-positive bacteria with disc diffusion method, *S. aureus* and *S. epidermidis* were most vulnerable to methanolic extract of *A. europaeum*. *S. aureus* was more sensitive to this extract (7z; 24 mm) than reference antibiotics lincomycin (21 mm), carbenicillin (18 mm), bacitracin (22 mm) and tetracycline (16 mm). Similarly, this extract showed better antibacterial activity (23.33 mm) than reference antibiotic bacitracin (14 mm) against *S. epidermidis* (Table 2). *S. pyogenes* was most susceptible to aqueous and ethanolic extracts of *D. orientale*, which have more inhibitory activity (20.67 mm and 19.33 mm, respectively) than tested antibiotics lincomycin (12 mm) and novobiocin (17 mm) (Table 2).

The well diffusion method demonstrated that although ethanolic extract of *A. europaeum* showed best antibacterial activity against *S. aureus*, methanolic extract of *A. europaeum* showed the best antibacterial activity against *S. epidermidis*. Also, ethanolic extract of *D. orientale* and aqueous extract of *D. pentaphyllum* exhibited strong inhibition against *S. pyogenes* (Table 3).

With regard to gram-negative bacteria with disc diffusion method, *P. aeruginosa* was most susceptible to ethanolic and methanolic extract of *A. mollis* and *D. pentaphyllum*, ethanolic extract of *E. campestre* and methanolic extract of *C. varia* and *R. angustifolius* (Table 2). Ethanolic and methanolic extracts of *A. mollis* (22.67 mm for both extracts) and ethanolic extract of *D. pentaphyllum* (22 mm) showed similar or statistically greater antibacterial activity than the reference antibiotics novobiocin (23 mm), carbenicillin (22 mm), tetracycline (13 mm), bacitracin (11 mm), ampicillin (8 mm) and lincomycin (7 mm). *K. pneumoniae* was most vulnerable to ethanolic and methanolic extracts of *R. angustifolius*, methanolic extract of *C. varia* and ethanolic extract of *C. olympica*. These extracts showed similar or greater inhibitory activity (between 21.33 and 20 mm) than the tested reference antibiotics carbenicillin (21 mm), ampicillin (17 mm), tetracycline (14 mm), novobiocin (12 mm), lincomycin (12 mm) and bacitracin (8 mm). *E. coli* showed best sensitivity to ethanolic extract of *E. campestre* and methanolic extract of *R. angustifolius* which had greater inhibitory activity (28.67 mm and 23.33 mm, respectively) than the reference antibiotics carbenicillin (21 mm), tetracycline (20 mm), novobiocin (17 mm), ampicillin (16 mm), bacitracin (9 mm) and lincomycin (8 mm) (Table 2).

The well diffusion method demonstrated that *P. aeruginosa* was most susceptible to ethanolic and methanolic extract of *A. mollis* and *D. pentaphyllum*. Aqueous and methanolic extract of *C. varia* showed best antibacterial activity against *K. pneumoniae*. *E. coli* showed best sensitivity to methanolic extract of *R. angustifolius* (Table 3).

Tested extracts of *E. campestre* exhibited antibacterial activity against *S. epidermidis*, *S. pyogenes*, *P. aeruginosa* and *E. coli* in our study (Tables 2 and 3). Ethanolic and methanolic extracts of *E. bithynicum* showed inhibition against only *S. pyogenes* [26] and no antibacterial activity was observed against five different fish pathogens (*Aeromonas hydrophila*, *Yersinia ruckeri*, *Streptococcus agalactia*, *Lactococcus garvieae* and *Enterococcus faecalis*),[26,27] which also recorded the antibacterial activity of *Eryngium foetidium* against *Helicobacter pylori*.

Benli et al. [28] reported the strong antibacterial activity of *Campanula lyrata* against *Baccillus subtilis* and *S. aureus*. In this study, all tested extracts of *C. glomerata* and *C. olympica* did not show any inhibitory activity against *S. aureus*, *S. epidermidis* and *P. aeruginosa*. These extracts displayed noticeable antibacterial activity against *K. pneumoniae* (Tables 2 and  3). Among the studied plant extracts, all tested extracts of *O. oxyodonta* and *F. pontica* had less activity against the used bacteria (Tables 2 and 3).

### SEM analysis at 24 h

**Figure 1.**
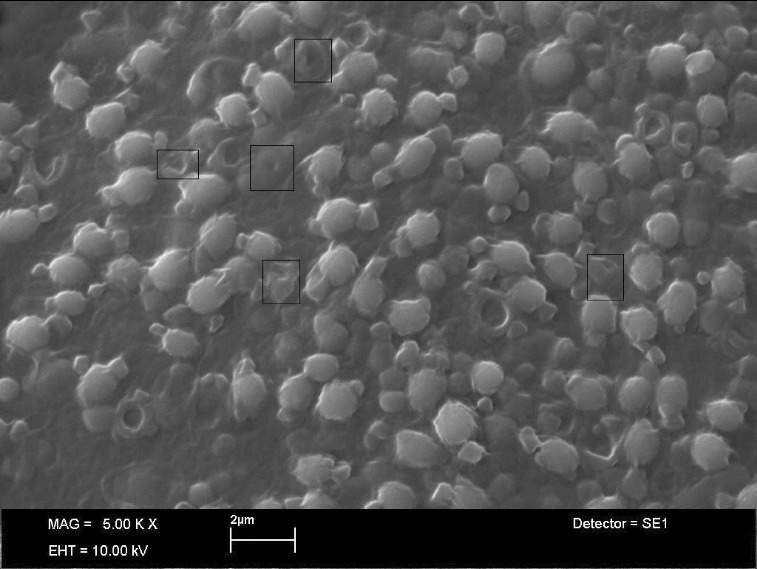
Scanning electron microscope images of *S. aureus* cells treated with methanol extract of *A. europaeum*. The morphologically changed cells were marked with the rectangles.

The SEM analysis after 24 h confirmed the effects of methanolic extract of *A. europaeum* on *S. aureus* cells. The surface changes of the bacterial cells were observed through the SEM images (Figure 1). The observable effects on the surface morphology of *S. aureus* bacteria during its logarithmic growth phase were displayed in Figure 1. According to the image, the treated bacterial cells appeared to be shrinking. Exposure to the extract resulted in occasional morphologic defects characterized by tubular outpouching of cell wall. Irregular spherical structures lying free or appearing to extrude from cells were also observed (Figure 1).

### Antitumour activity

Strong antitumour activity was observed with *A. europaeum* and *E. campestre* with *A. tumefaciens*-induced potato disc tumour assay. A prerequisite for this assay is that the extract or substance being tested should not have antibacterial activity toward *A. tumefaciens*.[25] Inhibition of crown gall formation on potato discs is caused by two effects: by antitumourogenesis or decreasing the viability of the *A. tumefaciens*. Viability tests were carried out with all extracts to distinguish between these possibilities. Bacterial viability was determined by incubating plant extracts with 1 × 10^3^ CFU of *A. tumefaciens* bacterial suspension and left for 30 min. As the attachment of the bacterium to a tumour-binding site is complete within 15 min following inoculation,[29] 30 min exposure was chosen in the experiment.[30] There was no difference in bacterial growth across the plates between control (only *A. tumefaciens*) and tested extracts (*A. tumefaciens* + plant extracts) in terms of colony counts (ranged from 9.2 × 10^3^ to 13 × 10^3^ CFU) except *A. mollis* and *D. pentaphyllum* extracts. All tested extracts other than *A. mollis* and *D. pentaphyllum* did not affect the viability of the bacterium. Thus, observed inhibition of tumour formation for these extracts was on the formation of tumours and not on the viability of the bacterium. On the other hand, *A. mollis* and *D. pentaphyllum* extracts affected the viability of the bacterium and *A. tumefaciens* bacterial growth was not observed across the plates. Therefore, it was understood that inhibition of crown gall formation on potato disc is caused by decreasing the viability of the *A. tumefaciens* for *A. mollis* and *D. pentaphyllum* extracts. Because of the strong antibacterial activity of *A. mollis* and *D. pentaphyllum* extracts against *A. tumefaciens*, it was not possible to evaluate the antitumour activity of these extracts with potato disc bioassay. Although the results herein did not prove an antitumour effects for the extracts of *A. mollis* and *D. pentaphyllum*, anticancer activity of these plants should be studied using different cancer cell lines. Strong antibacterial activity of *A. mollis* was also observed against *P. aeruginosa* in our study (Tables 2 and 3). High level of antibacterial activities of *A. mollis* may be due to its chemical composition including high level of total phenolics and condensed tannins.[31]

Best antitumour activity was obtained with aqueous extract of *A. europaeum* (100% tumour inhibition). Methanolic extract of *E. campestre* also exhibited very strong tumour inhibition (80.6% tumour inhibition) (Table 4). Other tested extracts of *E. campestre* (aqueous and ethanol) and *A. europaeum* (ethanol and methanol) also showed moderate level of antitumour activity (between 50% and 75% tumour inhibition). At least one extract of *C varia*, *O. oxyodonta*, *F. pontica*, *C. glomerata* and *C. olympica* have moderate level of antitumour activity (between 55.6% and 75% tumour inhibition). Least antitumour activities (less than 50% tumour inhibition) were obtained with all extracts of *R. angustifolius* and *D. orientale* (Table 4).

**Table 4.** Antitumour activity of used plant extracts. Means with the same letter within columns are not significantly different at *P* > 0.05.

Treatments	Mean no. of tumours (± SE)	% tumour inhibition
Water	35.75 ± 4.54 ^k^	–
Camptothecin	0 ± 0^a^	100
1x	14.08 ± 1.51 ^bcdef^	61.1
1Y	16.92 ± 1.84 ^cdefg^	52.8
1Z	6.75 ± 1.08^ab^	80.6
4X	30.17 ± 3.62 ^ijk^	16.7
4Y	11.92 ± 2.15 ^bcde^	66.7
4Z	30.33 ± 4.84 ^ijk^	16.7
5X	33.25 ± 3.59 ^ik^	8.3
5Y	29.5 ± 4.78 ^ijk^	16.7
5Z	12.17 ± 1.93 ^bcde^	66.6
6X	11.58 ± 1.63 ^bcd^	66.7
6Y	27.25 ± 3.81 ^hyk^	25.0
6Z	15.83 ± 2.19 ^bcdef^	55.6
7X	0 ± 0^a^	100
7Y	11.67 ± 2.44 ^bcd^	66.7
7Z	8.83 ± 2.26 ^bo^	75.0
8X	21.5 ± 2.52^el9hi^	38.9
8Y	30.75 ± 3.51 ^ijk^	13.9
8Z	23.42 ± 3.89 ^fghi^	36.1
9X	25.5 ± 3.33 ^ghii^	27.8
9Y	30.09 ± 3.77 ^ijk^	19.4
9Z	18.92 ± 2.23 ^defgh^	47.2
10X	18.5 ± 2.11 ^odelgh^	47.2
10Y	21.5 ± 3.13^elghi^	38.9
10Z	16.08 ± 1.71 ^bcdef^	55.6
11X	9.5 ± 1.28^bcd^	72.2
11Y	28.92 ± 3.4 ^ijk^	19.4
11Z	12.58 ± 1.44^bcde^	63.9

No tumour formation (100% tumour inhibition) (Table 4) was observed with aqueous extract of *A. europaeum* that was one of the best tested plants exhibiting strong antibacterial activities against all used bacteria except *S. pyogenes* in our study (Tables 2 and 3). Gracza [16] determined and evaluated the biologic activities (local anesthetic and expectorant) of the phenylpropanoids found in *A. eurapeum*. The phenylpropanoid ingredient of *A. europaeum* may contribute to the demonstrated strong antibacterial and antitumour activities.

Saponin,[5] coumarin [6]) and flavonoid [8] content of *E. campestre* may contribute to high level of antitumour (80.6% inhibition) and antibacterial activity (Tables 2– 4). Some studies [12,13] reported the antitumour activity of cardenolides obtained from ethanolic extract of *C. varia* seeds. In this study, ethanolic extract of aerial parts (flowers, leaves and stem) of *C. varia* showed moderate tumour inhibition (66.7%) (Table 4).

Since final concentrations of all extracts were adjusted with distilled water, it was used as a negative control and no inhibition was observed with water. Tumour formation was not observed with positive control camptothecin (100% inhibition).

The inhibition of *A. tumefaciens*-induced tumours (or crown gall) in potato disc tissue is an assay based on antimitotic activity and can detect a broad range of known and novel antitumour effects.[24,25] The validity of this bioassay is predicted on the observation that certain tumourigenic mechanisms are similar in plants and animals. It was demonstrated that inhibition of crown gall tumour initiation on potato disc showed an apparent correlation with compounds and plant extracts known to be active in the 3PS (*in vivo*, murine leukaemia) antitumour assay.[25,32] Ferrigini et al. [33] showed that crown gall tumours on potato discs could routinely be employed as comparatively rapid, inexpensive, safe, and statistically reliable prescreen for 3PS antitumour activity.

## Conclusions

Antibacterial and antitumour activities of 33 different extracts obtained from 11 different plants grown in Turkey were evaluated. Strong antibacterial activities were obtained with all tested extracts of *A. europaeum* against *S. aureus*. Alcoholic extracts of *E. campestre*, *A. mollis*, *D. pentaphyllum*, *C. varia*, *R. angustifolius*, *C. glomerata* and *C. olympica* also showed strong antibacterial activities against *E. coli*, *P. aeruginosa* or *K. pneumoniae*. Aqueous extract of *A. europaeum* and methanolic extract of *E. campestre* exhibited strong tumour inhibition. Тhese results show some scientific justification for the tested plants to be used as medicinal plants. In the future, identification of active components can be studied for plant extracts having strong bioactivity. Future studies should focus on fractionation of the extracts in hopes of identifying active components. Anticancer activity of these plants should be studied using different cancer cell lines in the future. 
